# Association of MTHFR, SLC19A1 Genetic Polymorphism, Serum Folate, Vitamin B_12_ and Hcy Status with Cognitive Functions in Chinese Adults

**DOI:** 10.3390/nu8100665

**Published:** 2016-10-24

**Authors:** Can Cai, Rong Xiao, Nicholas Van Halm-Lutterodt, Jie Zhen, Xiaochen Huang, Yao Xu, Shuying Chen, Linhong Yuan

**Affiliations:** 1 School of Public Health, Capital Medical University, Beijing 100069, China; ccfdz10223@163.com (C.C.); xiaor22@ccmu.edu.cn (R.X.); zhenjie66@ccmu.edu.cn (J.Z.); huangxiaocheng@126.com (X.H.); xuyao1992@ccmu.edu.cn (Y.X.); sycheng@ccmu.edu.cn (S.C.); 2 Department of Neurosurgery, Tiantan Hospital, Capital Medical University, Beijing 100050, China; NickVH@ccmu.edu.cn

**Keywords:** cognition, gene polymorphism, nutrition, geriatrics

## Abstract

Background/Aim: Studies have indicated a relationship between either gene polymorphism or in vivo B vitamins’ nutritional status with cognition in the elderly. However, the combined effects of MTHFR and SLC19A1gene polymorphism with serum folate and vitamin B_12_ levels on cognition in Chinese adult population remain unclear. Methods: Demographic information of 426 Chinese adults aged from 55 to 90 were collected by a well designed self-administered questionnaire. The Montreal Cognitive Assessment test was utilized to evaluate the cognition status of the participants. MTHFR and SLC19A1 genotyping was analyzed using polymerase chain reaction-ligase detection reaction (PCR- LDR) method. Serum folate, vitamin B_12_ and homocysteine (Hcy) levels were detected by commercial assay kits. Pearson’s correlation was used for data analyses and statistical significance was set at *p* < 0.05. Results: Serum Hcylevels demonstrated a negative correlation with serum folate (*r* = −0.301) and vitamin B_12_ (*r* = −0.292) levels. The negative correlation found between serum Hcy levels and attention ability was observed in all 426 studied subjects (*r* = −0.122). Subjects with MTHFR 677 T/T and 1298 A/A genotypes demonstrated a higher serum Hcy levels (*p* < 0.05). Carriers of MTHFR (1298 A/C + C/C and 1793 G/A) and SLC19A1 80 G/G genotypes showed lower abstraction and delayed memory ability, respectively (*p* < 0.05). Subjects with MTHFR 1793 G/A genotype along with low serum folate concentration demonstrated the lowest name and orientation abilities. The effects of MTHFR 1793 G/A genotype on cognitive performance were dependent on the status of serum vitamin B_12_. Conclusion: Cognition of adults was associated with MTHFR, SLC19A1 gene polymorphism and serum Hcy levels. This study clearly establishes a combined effect of MTHFR gene polymorphism and serum B vitamins levels on cognition in Chinese adults.

## 1. Introduction

Alzheimer’s disease (AD) is recognized as the most common entity of dementia in the elderly. Mild cognitive impairment (MCI) is regarded as an early stage of AD, which results in memory loss beyond the expected for age and learning [1,2,3]. It is further reported that this brain degenerative process of dementia is inevitably irreversible [4]. The pathogenesis of AD still stands as foggy and, till date, there is no available specific and/or effective strategy for the prevention and treatment of this neuro-degenerative disorder [5,6]. Hence, it is critically essential to identify the potential risk factors and biomarkers for the diagnoses of MCI and AD in the elderly. 

Folate and vitamin B_12_ are well acknowledged as essential nutrients that play key roles in the normal functions of the brain. The deficiency of folate and vitamin B_12_ has been commonly reported in the elderly [7,8]. The synthesis of methionine from homocysteine (Hcy), catalyzed by methionine synthase, needs an interaction between folate and vitamin B_12_ [9]. Therefore, serum Hcy levels are largely determined by the in vivo folate and vitamin B_12_ nutritional status [10]. A cross-sectional study observed a negative correlation between circulating folate and vitamin B_12_ status with serum Hcy levels [11]. Moreover, elderly subjects with optimum serum folate and vitamin B_12_ levels have been shown to perform well in certain specific cognitive domains [12]. A recent clinical trial has demonstrated that treatment with B-group vitamins significantly halted the progression from MCI to AD [13]. This evidently implies that low serum folate and vitamin B_12_ levels and elevated Hcy levels might be potential risk factors contributing to the development of MCI and AD in the elderly. 

There is a wide variation in the prevalence of methylenetetrahydrofolate reductase (MTHFR) and reduced folate carrier (SLC19A1/RFC1) gene polymorphism across different populations around the world. MTHFR and SLC19A1/RFC1 gene products have been reported to be involved in the biological metabolism of folate, vitamin B_12_ and Hcy, maintaining DNA methylation patterns by donating carbon atoms [14]. Given the role of brain DNA methylation in memory and AD [15], confirmation of the epigenetic impacts associated with MTHFR and SLC19A1/RFC1 gene mutations as markers for MCI or AD could open new potential research domains for the prevention and treatment of dementia in the elderly [16].

Recently, the effects of genetic polymorphism of MTHFR and SLC19A1 genes on circulating folate, vitamin B_12_, Hcy levels and cognition have aroused particular attention. However, the conclusions still remain controversial [17,18,19]. Moreover, limited studies have explored the interactions of serum folate, vitamin B_12_ and Hcy levels with respect to MTHFR and SLC19A1 gene polymorphism on cognitive function in the elderly. Therefore, in the current study, we carried out a community-based cross-sectional study aiming to explore the relationship of serum folate, vitamin B_12_ and Hcy levels with MTHFR and SLC19A1 genetic polymorphism and cognition in Chinese adults. The results of the present study will provide a theoretical basis and foundation for uncovering the potential interactions that exist between nutritional and genetic backgrounds of cognition in adults. 

## 2. Materials and Methods

### 2.1. Participants

Participants aged 55–90 were randomly recruited from Nanyuan Community (Beijing, China) by posting advertisements and phone calls. Data were collected between May 2013 and July 2014. Exclusion criteria were severe diseases or conditions known to affect cognitive function (e.g., inflammatory diseases, recent history of heart or respiratory failure, chronic liver disease or renal failure, malignant tumors, a recent history of alcohol abuse, history of cerebral apoplexy or cerebral infarction). The subjects with AD, Parkinson’s disease (PD), long-term frequency intake of antidepressants and medication acting on central nervous system, or those unable to finish the cognition tests were also culled from the study. In total, 475 adults participated in the study, while 49 subjects were excluded due to uncompleted questionnaires or unsuccessful genotyping. The Medical Ethics Committee of Capital Medical University (No. 2012SY23) approved the study and written informed consents were obtained from all participants. 

### 2.2. Anthropometric Measurements and Socio-Demographic Variables

Anthropometric parameters (height and weight) were measured by nurses from the community’s health service center. Body mass indices (BMIs) were calculated as weight (kg)/height (m^2^). Information on demographic characteristics (e.g., age, gender, nationality, and education), lifestyle factors (e.g., living condition (living alone, yes or no), smoking (non-smoker or current smoker), alcohol drinking (yes or no), physical activity (no physical activity or exercise regularly), reading (reading regularly or never), TV viewing or computing (everyday or never) and housekeeping (everyday or never)), medical history of chronic diseases and dietary supplements were collected by self-administered questionnaires. Education level was assessed as the highest level attained and classified into six categories (illiterate, primary school, junior high school, high school, junior college, undergraduate and above). 

### 2.3. Cognitive Tests

Cognitive function was assessed by Montreal Cognitive Assessment (MoCA), which consists of seven cognitive domains including visual-spatial and executive ability, namely, attention, attraction, language, delayed memory and orientation functions. The MoCA appears to have utility as a cognitive screening tool with high sensitivity and specificity for early detection of MCI and AD [20,21]. According to a previous study conducted in elderly Chinese population, the cut-off points used for MCI diagnosis were as follows: 13/14 for individuals with no formal education, 19/20 for individuals with 1 to 6 years of education, and 24/25 for individuals with 7 or more years of education. The cut-offs above were shown to be sensitive and efficient in the diagnosis of MCI in an elderly Chinese population [22]. In the current study, the test was carried out by trained investigators in the Nanyuan Community Health Service Center. 

### 2.4. Blood Measurement

#### 2.4.1. Measurement of Plasma Parameter

Fasting venous blood samples was collected between 8:00 a.m. and 9:00 a.m. from each subject. For plasma parameter determination, blood samples were centrifuged in lithium heparin tubes at 480 g for 10 min at 4 °C, and then stored at −20 °C before further analyses. Plasma glucose (Glu), triglyceride (TG) and total cholesterol (TC) were measured by an ILAB8600 clinical chemistry analyzer (Instrumentation Laboratory Lexington, Lexington, WI, USA). A commercially available assay from Instrumentation Laboratory was used to determine high density lipoprotein cholesterol (HDL-C). Low density lipoprotein cholesterol (LDL-C) was calculated using the Friedewald formula [23]. All samples of each subject were analyzed within a single batch, and the inter-assay coefficients of variation (CV) for all determinations were less than 5%. 

#### 2.4.2. Measurement of Serum Vitamin and Hcy Level Vitamin B_12_

Serum folate concentration was measured by an assay kit purchased from R-Biopharm AG (Darmstadt, Germany) according to the manufacturer’s instruction. Was analyzed by chemiluminescence method (Abbott Laboratories Ltd., Lake Bluff, IL, USA). Besides, Hcy was detected using turbidimetric inhibition immunoassay by an AU480 automatic biochemical analyzer (Olympus, Tokyo, Japan). Three independent measurements were performed for each sample. 

#### 2.4.3. Genotyping

Genomic DNA was extracted from peripheral blood leukocytes using the Wizaregenomic DNA purification kit (Promega, Madison, WI, USA). MTHFR C677T (rs1801133), MTHFR A1298C (rs1801131), MTHFR G1793A (rs2274976), and SLC19A1 G80A (rs1051266) polymorphisms were genotyped by a patented multiplex LDR method (iMLDR, Genesky Bio-Tech Cod., Ltd., Shanghai, China) [24], with technical support from the Shanghai Genesky Biotechnology Company (Shanghai, China). Finally, the samples were analyzed by the ABI3130XL sequencer (Applied Biosystems, Foster City, CA, USA) and the raw data were analyzed by GeneMapper4.1 (Applied Biosystems, Foster City, CA, USA). In addition, 20% of DNA samples were genotyped again by different operations for the purpose of quality control of the genotyping. 

### 2.5. Statistical Analyses

Data analysis was carried out using SPSS 19.0 (SPSS Inc., IBM Corporation, Chicago, IL, USA). Continuous variables including age, BMI, blood parameters (folate, vitamin B_12_, Hcy, GLU, TC, TG, HDL-C, and LDL-C) and MoCA score were presented as mean (95% confidence interval, CI). Gender, nationality, education level, living condition, smoking habit, alcohol drinking, physical activity, reading, television viewing or computing, housekeeping and genetic polymorphism in MTHFR and SLC19A1 were presented as category variables. Pearson correlation coefficients were presented to describe the association between serum folate, vitamin B_12_, Hcy levels and cognitive function. Participants were classified according to MTHFR or SLC19A1 genotypes and median concentrations of serum folate, vitamin B_12_ and Hcy. General linear model (GLM) was applied for the analysis of the difference among groups and Bonferroni correction for multiple comparisons. For folate, vitamin B_12_ and Hcy levels, some potential confounding factors including gender, age and BMI were adjusted for feasibility enhancement in data analyses. For cognition analysis, factors including gender, age, BMI, education, living condition, reading and smoking habit were adjusted. Statistical significance was set at *p* < 0.05.

## 3. Results

### 3.1. Demographic Characteristics of the Participants

The distributions of demographic characteristics of the participants are presented in Table 1. Totally, there were 118 (27.7%) male and 308 (72.3%) female included in this present study. The average age of the subjects was 62.61 years. The mean BMI of the subjects was 25.58 kg/m^2^. A majority of the participants had an educational level of junior high school or above (71.9%). Only 32 (7.5%) of participants lived alone. In total, 37.8% subjects of all the participants demonstrated the habit of reading. The subjects with smoking or alcohol drinking habit accounted for 14.3% and 21.8%, respectively, of all participants. The average serum folate, vitamin B_12_ and Hcy levels were 9.37 (8.83, 9.96) ng/mL, 511.44 (275.29, 547.55) pg/mL and 13.50 (12.85, 14.25) μmol/L, respectively. Among the 426 subjects, nearly half were heterozygote variants of MTHFR C677T C/T (46.7%) and SLC19A1 G80A G/A (50.2%). Majority of them were homozygotes of MTHFR G1793A G/G (90.8%) and MTHFR A1298C A/A (76.5%). However, only nine (2.1%) participants were MTHFR A1298C C/C genotype. Thus, the carriers of one or two copies of the C allele were pooled during the statistical analysis. 

### 3.2. Association among Folate, Vitamin B_12_, Hcy and Cognitive Function

As shown in Table 2, a significant positive correlation between serum folate and vitamin B_12_ concentrations was detected (*r* = 0.171, *p* < 0.05). Serum folate and vitamin B_12_ levels were negatively correlated with Hcy content in serum (*r*_foate_ = −0.301, *p* < 0.01; *r*_vitmain B12_ = −0.292, *p* < 0.01). Negative correlation between serum Hcy content and attention ability were observed in the studied population (*r* = −0.122, *p* < 0.05). Nevertheless, we did not detect any relationship between cognitive functions (MoCA scores) and serum folate or vitamin B_12_ levels (*p* > 0.05). 

### 3.3. Folate, Vitamin B_12_ and Hcy According to Genetic Polymorphism

No association with MTHFR and SLC19A1 polymorphism and serum folate or vitamin B_12_ levels was observed. Comparing with carriers of MTHFR 677C allele (C/C and C/T), subjects with MTHFR 677 T/T genotype had higher serum Hcy level (*p* < 0.01). Subjects with MTHFR 1298 homozygote (C/C) and heterozygote variants (A/C) had lower serum Hcy concentration than subjects with normal homozygotes (A/A) (*p* < 0.05). Serum Hcy concentration of the participants was not MTHFR G1793A and SLC19A1 polymorphisms associated (Table 3). 

### 3.4. Cognition According to Folate, Vitamin B_12_, Hcy and Genetic Polymorphism

No associations of MTHFR C677T polymorphism and serum folate, vitamin B_12_ and Hcy levels with cognitive performance were detected (Tables S1–S3). As shown in Table 4, carriers of MTHFR 1298 A/C + C/C genotype had lower abstraction ability as comparing with subjects with MTHFR 1298 A/A genotype (*p* < 0.05). However, no effect of MTHFR A1298C polymorphism on other cognitive domains was observed (*p* > 0.05). In addition, no combined effects of MTHFR A1298C polymorphism and serum folate, vitamin B_12_ and Hcy levels on cognition were observed (*p* > 0.05) (Tables S4–S6). 

Individuals with MTHFR 1793 G/A genotype had lower abstraction scores than those with MTHFR 1793 G/G genotype (*p* < 0.05). After categorizing the participants with MTHFR G1793A genotypes and the serum folate concentration, carriers of MTHFR 1793 G/A genotype along with low serum folate concentration (below the median) had the lowest scores in name and orientation abilities. No significant combined effects of MTHFR G1793A polymorphism and serum folate level on other cognitive domains were observed (*p* > 0.05) (Table 5). In addition, under low serum vitamin B_12_ threshold condition (below the median), cognitive performance on the attention, abstraction and orientation abilities as well as total MoCA scores were found to be lower in the subjects with G/A genotype as compared to the subjects of the G/G genotype for MTHFR 1793 polymorphism (*p* < 0.05). Besides, there was a significant combined effect of MTHFR G1793A polymorphism and serum vitamin B_12_ level on abstraction ability. The association of MTHFR 1793 polymorphism with abstraction ability was undetectable in the subjects with higher serum vitamin B_12_ concentration (above the medium level). When serum vitamin B_12_ level was below the median, the subjects with G/G genotype had a 23% and 13% increase in abstraction scores and total MoCA score, respectively, relative to the subjects with G/A genotype (*p* < 0.05) (Table 6). No association of MTHFR G1793A polymorphism and serum Hcy level with cognitive function was detected in the current study (*p* > 0.05) (Table 7). 

The association of SLC19A1 G80A polymorphism with cognition was shown in Table 8. Comparing with carriers of 80A allele of SLC19A1 (G/A and A/A), subjects with SLC19A1 80 G/G genotype had lower delayed memory scores (*p* < 0.05). However, no association of SLC19A1 G80A polymorphism, serum folate, vitamin B_12_ and Hcy levels with cognition was observed (*p* > 0.05) in the investigated population (Tables S7–S9). 

## 4. Discussion

To our knowledge, this present study is the first to explore the relationship between serum folate, vitamin B_12_, Hcy levels, folate metabolism related enzyme gene polymorphisms and cognitive function in Chinese adults. Our results clearly establish the negative correlation between serum B vitamin and Hcy levels in indicated participants (Table 2). We also detected the association of MTHFR or SLC19A1 genetic polymorphism with circulating Hcy concentration or cognitive function in the investigated Chinese adults. The modifying effects of serum folate and vitamin B_12_ levels on the relationship between MTHFR polymorphism and cognition were observed. 

High serum Hcy content, caused by deficiency of B vitamins has been reported to be associated with the risk of AD in population based studies [25,26,27,28]. Gorgone and coworkers found that hyperhomocysteinemia damaged cognitive function via micro-vascular damage and direct neuro-toxic effect [29]. Furthermore, increasing published studies indicate that elevated blood Hcy level is recognized as a risk factor for dementia and cognition decline in the elderly [30,31]. In this present study, we detected a relationship between hyperhomocysteinemia and the decline of cognitive performance (attention domain). Tucker and coworkers reported that lower serum B_12_ vitamin and higher homocysteine concentrations predict cognitive decline. A higher homocysteine concentration has also been associated with a decline in recall memory [32]. Interestingly, according to data from the third National Health and Nutrition Examination Survey in America, hyperhomocysteinemia demonstrated a correlation to poor recall ability independent of circulating folate status in subjects aged 60 years or above [33]. Another study’s data from the cross-sectional analyses carried out by Mooijaart et al. further indicated that serum concentration of homocysteine was significantly associated with cognitive performance in older adults. They also discovered that serum concentration of homocysteine was inversely linked with cognitive impairment in the elderly [34]. Together with these findings, our results suggest that serum Hcy and B_12_ vitamin concentrations might be potential predictors of cognitive impairment [35,36]. 

In the present study, we did not observe the relationship between SLC19A1 polymorphism and circulating Hcy concentration in the detected subjects. This result was in accordance with the findings of a population-based study in Dutch carried out by Lonneke and coworkers [37]. However, we observed that subjects with homozygote (T/T) in MTHFR C677T had significantly higher serum Hcy levels compared to 677C carriers. They also found that higher Hcy concentration was found in subjects with MTHFR A1298C AA homozygote as compared with MTHFR A1298C A/C + C/C genotypes carriers. Similar results were also reported by Zappacosta and coworkers’ cross-sectional study carried out in mid-southern Italy [38]. The authors observed that subjects with MTHFR A1298C wild-type had higher serum Hcy concentration. It was generally recognized that MTHFR gene products involved in a central reaction in folate metabolism, catalyzed the synthesis of 5-methyltetrahydrofolate from 5,10-methylenetetrahydrofolate [39]. The enzyme activity could be affected by the mutations of MTHFR gene resulting in abnormal folate metabolism and indirect hyperhomocysteinemia. However, this result is contradictory to other previous studies. Summers et al. reported that MTHFR 1298C carriers had a higher plasma Hcy concentration than 1298AA homozygotes after the exclusion of MTHFR 677TT homozygotes in Caucasian premenopausal women rather than African-American [40]. Another cross-sectional study carried out in four eastern states of India observed similar patterns in Healthy population [41]. The inconsistency in regard to the results of these published studies could be attributed to the geographical differences in populations and physiological statuses. Therefore, further research is needed to uncover the relationship(s) between MTHFR A1298C polymorphism and circulating Hcy concentrations in the elderly. 

Research aiming to explore the association between MTHFR G1793A genetic polymorphism and specific cognitive domains appears to be currently lacking. In the current study, we did not observe the effect of MTHFR C677T polymorphism on cognition (MoCA score) in Chinese adults. This direction was adopted based on the findings of Polito et al. [42]. In their study, the authors found out MTHFR C677T genotypes were not associated with cognitive performance. Additionally, we found that carriers of MTHFR 1298 A/C + C/C and 1793 G/A genotypes had lower scores on abstraction ability as compared to subjects with MTHFR 1298 A/A and 1793 G/G genotypes. A large meta-analysis of genetic studies and clinical trials indicate that the effect of MTHFR genotype on the risk of chronic disease (such as stroke) needs to be assessed in the context of baseline folate levels. Huo’s study found that the highest risk of stroke and the greatest benefit of folic acid therapy were found in subjects with the MTHFR C677T CC or CT genotypes and the lowest baseline folate levels [43]. These results reflect the combined effects of MTHFR gene polymorphism and in vivo folate nutritional status on cognition. Malaguarnera and coworkers reported that inadequate serum folate and vitamin B_12_ status were significantly associated with AD [44]. An interesting finding observed in the present study is that the serum folate and vitamin B_12_ levels dictated a dependent influence on MTHFR 1793 G/A genotypes associated with cognitive function in Chinese adults (Table 5 and Table 6). The adults with MTHFR 1793 G/A genotype and low serum folate concentration (below the median) exhibited poor cognitive performance in name and orientation domains. Cognitive performance of attention, abstraction and orientation as well as total MoCA scores was significantly lower in carriers of MTHFR 1793 G/A genotype along with low vitamin B_12_ levels (below the median). These results imply that lower serum folate or vitamin B_12_ status might expose subjects with MTHFR 1793 G/A genotypes to cognitive decline (especially, with the abilities of name, attention, abstraction and orientation), while, for the subjects with higher serum folate and vitamin B_12_ levels, the association of MTHFR 1793 G/A genotypes on cognitive function was undetectable. These data demonstrated the combined effects of serum folate, vitamin B_12_ levels and MTHFR G1793A polymorphism on cognitive function. It was well known that adequate amounts of circulating folate and vitamin B_12_ levels might decrease the risk of cognitive impairment [45]. Therefore, an optimum intake of folate and vitamin B_12_ might be necessary for older adults to keep normal cognition, especially, the MTHFR 1793 G/A genotype carriers.

In this current study, we further explored the association of serum folate, vitamin B_12_, Hcy levels and SLC19A1 G80A genotypes on cognition of the elderly. As shown in Table 8, we only detected the genotype differences of delayed memory ability in the investigated participants. Outcomes show that subjects with SLC19A180G/G genotypes seemed to have higher risk of delayed memory ability impairment compared to subjects with other SLC19A1 genotypes. A case-control study conducted by Bi et al. in Beijing revealed that SLC19A1 80G alleles and 80G/G genotype were significantly associated with the risk of sporadic AD and SLC19A1 80G alleles was observed as an ApoEepsilon4 independent risk factor for late-onset AD [46]. In the current study, we found a beneficial effect of 80A alleles of SLC19A1 on delayed memory performance. Nevertheless, a case-control study carried out by Mansoori and coworkers did not indicate the direct association between SLC19A1 G80A genotypes and AD [47]. Bialecka and coworkers reported that polymorphism of SLC19A1 was not associated with cognitive impairment in patients with Parkinson’s disease (PD) [17]. This further implies that a large scale cohort studies is essentially needed to ascertain the relationship between SLC19A1 polymorphism and cognition. 

Some limitations are considered in this present study. Firstly, our study was a community-based cross-sectional study, lacking the capacity of causal inference. Therefore, additional large scale cohort studies are needed to elucidate the effect(s) of serum folate, vitamin B_12_, Hcy and the genetic polymorphism of MTHFR and SLC19A1 on cognitive function in adults. Secondly, attributed to the different lifestyle and diet patterns, the circulating vitamin levels of participants in present study might be different from that of individuals in western countries. Besides, the distribution of MTHFR and SLC19A1 genotypes might be associated with differences in ethnic background [48]. As a result, the extrapolation of the conclusions to other populations should be considered with caution. Finally, after adjusting several common confounding factors during data analyses, our results might still be fraught with some influential or uncontrollable factors. Therefore, further follow-up strategies are required to clarify and replicate the results. 

## 5. Conclusions

In conclusion, our data evidently indicate a relationship between body folate and vitamin B_12_ nutritional status, MTHFR polymorphism and serum Hcy content in Chinese adults. Elevated serum Hcy levels might be a contributing factor to the decline of attention ability in the elderly. Abstraction and delayed memory abilities were MTHFR and SLC19A1 genotype-associated. The association of MTHFR G1793A polymorphism associated with cognition was serum vitamin B_12_ and folate levels dependent. 

## Figures and Tables

**Table 1:** Demographic characteristics of the participants.

Characteristics	Total (*n* = 426)	Characteristics	Total (*n* = 426)
Demographic variables, *n* (%)		Hcy (μmol/L)	13.50 (12.9, 14.3)
Age, mean (95% CI)	62.61 (62.03, 63.21)	GLU (mmol/L)	6.28 (6.1, 6.5)
Male gender	118 (27.7)	CHOL (mmol/L)	5.23 (5.1, 5.3)
BMI, mean (95% CI)	25.58 (25.24, 25.90)	TG (mmol/L)	2.14 (2.0, 2.3)
Han nationality	404 (94.8)	HDL-C (mmol/L)	1.41 (1.4, 1.4)
Education		LDL-C (mmol/L)	3.0 (2.9, 3.1)
Illiterate	27 (6.3)	Gene variants, *n* (%)	
Primary school	93 (21.8)	MTHFR C677T	
Junior high school	188 (44.1)	C/C	67 (15.7)
High school	99 (23.2)	C/T	199 (46.7)
Junior college	13 (3.1)	T/T	160 (37.6)
Undergraduate and above	6 (1.4)	MTHFR A1298C	
Living alone	32 (7.5)	A/A	326 (76.5)
Smoking	61 (14.3)	A/C	91 (21.4)
Drinking	93 (21.8)	C/C	9 (2.1)
Physical activity	389 (91.3)	MTHFR G1793A	
Reading	161 (37.8)	G/G	387 (90.8)
TV viewing or computing	416 (97.7)	G/A	39 (9.2)
Housekeeping	396 (93.0)	SLC19A1 G80A	
Plasma parameters, mean (95% CI)		G/G	145 (34.0)
Folate (ng/mL)	9.37 (8.8, 10.0)	G/A	214 (50.2)
Vitamin B_12_ (pg/mL)	511.44 (275.3, 547.6)	A/A	67 (15.7)

Abbreviations: BMI, body mass index; Hcy, homocysteine; GLU, glucose; CHOL, cholesterol; TG, triglyceride; HDL-C, high density lipoprotein cholesterol; LDL-C, low density lipoprotein cholesterol; MTHFR, methylenetetrahydrofolate reductase; SLC19A1, RFC (reduced folate carrier); CI, confidence interval.

**Table 2:** Association among serum folate, vitamin B_12_, Hcy status and cognitive function (*n* = 426).

Parameters and Cognition	Folate	Vitamin B_12_	Hcy
Parameters			
Folate	-	0.171 *	−0.301 *
Vitamin B_12_	0.171 *	-	−0.292 *
Hcy		−0.292 *	-
Cognition			
Visual-spatial and executive ability	0.001	0.015	−0.018
Name	−0.023	0.006	0.054
Attention	0.017	0.031	−0.122 *
Language	−0.040	−0.045	−0.038
Abstraction	−0.082	−0.004	−0.030
Delayed memory	0.011	0.050	−0.092
Orientation	−0.072	−0.012	0.048
MoCA score	−0.026	0.016	−0.420

Pearson correlation coefficients were presented to describe the association between variables. Hcy, homocysteine; MoCA, Montreal Cognitive Assessment. * *p* < 0.05.

**Table 3:** Serum folate, vitamin B_12_ and Hcy levels according to MTHFR and SLC19A1genotypes (*n* = 426).

Genotypes	Parameters		
	Folate (ng/mL)	Vitamin B_12_ (pg/mL)	Hcy (μmol/L)
MTHFR C677T			
C/C (*n* = 67)	9.59 (8.12, 11.06)	513.57(419.73, 607.41)	11.12 (9.58, 12.66) ^a^
C/T (*n* = 199)	9.29 (8.44, 10.14)	527.00 (471.89, 582.11)	12.18 (11.28, 13.08) ^a^
T/T (*n* = 160)	9.23 (8.28, 10.17)	491.29 (429.93, 552.66)	16.16(15.16, 17.17) ^b^
MTHFR A1298C			
A/A (*n* = 326)	9.26 (8.60, 9.92)	510.90 (467.87, 553.92)	14.08 (13.35, 14.82) *
A/C + C/C (*n* = 100)	9.49 (8.29, 10.68)	513.15 (436.52, 589.79)	11.66 (10.35, 12.97) *
MTHFR G1793A			
G/G (*n* = 387)	9.31 (8.71, 9.91)	510.34 (471.09, 549.59)	13.70 (12.98, 14.34)
G/A (*n* = 39)	9.37 (7.48, 11.27)	522.16 (399.55, 644.78)	11.96 (9.85, 14.08)
SLC19A1 G80A			
G/G (*n* = 145)	9.85 (8.87, 10.83)	463.77 (399.95, 527.60)	13.90 (12.79, 15.00)
G/A (*n* = 214)	9.28 (8.47, 10.09)	549.85 (498.13, 602.56)	13.16 (12.25, 14.08)
A/A (*n* = 67)	8.25 (6.80, 9.71)	490.51 (393.24, 587.78)	13.75 (12.07, 15.43)

General linear model was applied for the analysis of the difference among groups and Bonferroni correction for multiple comparisons. Factors including gender, age and BMI were adjusted for feasibility during data analyses. The data were presented as their obtained means with significance set at 95% CI. Hcy, homocysteine; MTHFR, methylene tetrahydrofolate reductase; SLC19A1, RFC (reduced folate carrier). *p* value < 0.05 was considered as significance. *: *p* < 0.05; ^a,b^: significantly different for multiple comparisons among different MTHFR C677T genotypes, *p* < 0.05.

**Table 4:** MoCA scores by MTHFR A1298C genotypes.

Cognition	Genotypes	MoCA Scores
Visual-spatial and executive ability	A/A (*n* = 326)	3.65 (3.51, 3.79)
	A/C + C/C (*n* = 100)	3.40 (3.14, 3.66)
Name	A/A (*n* = 326)	2.86 (2.80, 2.93)
	A/C + C/C (*n* = 100)	2.85 (2.73, 2.96)
Attention	A/A (*n* = 326)	5.12 (4.97, 5.26)
	A/C + C/C (*n* = 100)	5.11 (4.84, 5.38)
Language	A/A (*n* = 326)	2.08 (1.98, 2.17)
	A/C + C/C (*n* = 100)	2.00 (1.83, 2.17)
Abstraction	A/A (*n* = 326)	1.59 (1.51, 1.67) *
	A/C + C/C (*n* = 100)	1.41 (1.27, 1.56) *
Delayed memory	A/A (*n* = 326)	2.81(2.66, 2/97)
	A/C + C/C (*n* = 100)	2.73 (2.45, 3.01)
Orientation	A/A (*n* = 326)	5.69 (5.56, 5.82)
	A/C + C/C (*n* = 100)	5.60 (5.37, 5.84)
MoCA score	A/A (*n* = 326)	24.72 (24.12, 25.33)
	A/C + C/C (*n* = 100)	24.01 (22.90, 25.11)

Participants were classified according to the categories of MTHFR A1298C genotypes. The Data were reported as means (95% CI). General linear model was applied for the analysis. Factors including gender, age, BMI, education, living condition, reading and smoking habit were adjusted during the data analyses. MoCA, Montreal Cognitive Assessment; MTHFR, methylenetetrahydrofolate reductase. *p* value < 0.05 was considered statistically significant with asterisk superscript (*): *p* < 0.05.

**Table 5:** MoCA scores by serum folate level and MTHFR G1793A genotypes.

Cognition	Genotypes	Folate (ng/mL)		All Subjects
		≤7.49	>7.49	
Visual-spatial and executive ability	G/G (*n* = 387)	3.66 (3.50, 3.81)	3.59 (3.40, 3.78)	3.62 (3.49, 3.75)
	G/A (*n* =39)	3.33 (2.80, 3.85)	3.43 (2.85, 4.01)	3.35 (2.94, 3.77)
Name	G/G (*n* = 387)	2.94 (2.89, 2.98) *	2.80 (2.70, 2.90)	2.87 (2.81, 2.93)
	G/A (*n* = 39)	2.66 (2.50, 2.82) *	2.89 (2.59, 3.20)	2.75 (2.57, 2.93)
Attention	G/G (*n* = 387)	5.18 (5.03, 5.33)	5.08 (4.86, 5.30)	5.14 (5.01, 5.28)
	G/A (*n* = 39)	5.00 (4.49, 5.50)	4.93 (4.26, 5.60)	4.82 (4.40, 5.24)
Language	G/G (*n* = 387)	2.16 (2.05, 2.26)	2.01 (1.89, 2.13)	2.07 (1.99, 2.16)
	G/A (*n* = 39)	2.06(1.72, 2.41)	1.87 (1.49, 2.24)	1.93 (1.66, 2.20)
Abstraction	G/G (*n* = 387)	1.67 (1.58, 1.75)	1.46 (1.35, 1.57)	1.57 (1.50, 1.64)^*^
	G/A (*n* = 39)	1.49 (1.19, 1.78)	1.15 (0.82, 1.49)	1.35 (1.09, 1.55)^*^
Delayed memory	G/G (*n* = 387)	2.95 (2.76, 3.13)	2.65 (2.44, 2.85)	2.82 (2.68, 2.97)
	G/A (*n* = 39)	2.99 (2.39, 3.60)	2.28 (1.65, 2.90)	2.51 (2.02, 2.95)
Orientation	G/G (*n* = 387)	5.88 (5.79, 5.98) *	5.52 (5.31, 5.72)	5.70 (5.58, 5.82)
	G/A (*n* = 39)	5.39 (5.07, 5.71) *	5.48 (4.86, 6.09)	5.35 (4.98, 5.72)
MoCA score	G/G (*n* = 387)	25.36 (24.83, 25.89)	24.01 (23.09, 24.93)	24.72 (24.17, 25.28)
	G/A (*n* = 39)	23.79 (22.01, 25.56)	22.96 (20.16, 25.76)	22.92 (21.18, 24.67)

Participants were classified according to categories of MTHFR G1793A genotypes and serum folate concentration. The data were presented as means (95% CI). General linear model was applied for analysis. Factors including gender, age, BMI, education, living condition, reading and smoking habit were adjusted during the data analysis. MoCA, Montreal Cognitive Assessment; MTHFR, methylene tetrahydrofolate reductase. *p* value < 0.05 was considered statistically significant with asterisk superscript (*): *p* < 0.05.

**Table 6:** MoCA scores by serum vitamin B_12_ level and MTHFR G1793A genotypes.

Cognition	Genotypes	Vitamin B_12_ (pg/mL)		All Subjects
		≤412.5	>412.5	
Visual-spatial and executive ability	G/G (*n* = 387)	3.63 (3.45, 3.80)	3.62 (3.45, 3.80)	3.62 (3.49, 3.75)
	G/A (*n* = 39)	3.44 (2.88, 401)	3.32 (2.76, 3.88)	3.35 (2.94, 3.77)
Name	G/G (*n* = 387)	2.87 (2.80, 2.95)	2.86 (2.78, 2.94)	2.87 (2.81, 2.93)
	G/A (*n* = 39)	2.71 (2.46, 2.96)	2.85 (2.60, 3.11)	2.75 (2.57, 2.93)
Attention	G/G (*n* = 387)	5.13 (4.94, 5.31) *	5.14 (4.96, 5.33)	5.14 (5.01, 5.28)
	G/A (*n* = 39)	4.46 (3.87, 5.06) *	5.42 (4.83, 6.01)	4.82 (4.40, 5.24)
Language	G/G (*n* = 387)	2.14 (2.03, 2.26)	2.03 (1.92, 2.15)	2.07 (1.99, 2.16)
	G/A (*n* = 39)	1.82 (1.45, 2.19)	2.06 (1.70, 2.42)	1.93 (1.66, 2.20)
Abstraction	G/G (*n* = 387)	1.59 (1.49, 1.69) *	1.54 (1.44, 1.64)	1.57 (1.50, 1.64) *
	G/A (*n* = 39)	1.22 (0.90, 1.54) *	1.39 (1.07, 1.70)	1.35 (1.09, 1.55) *
Delayed memory	G/G (*n* = 387)	2.78 (2.58, 2.98)	2.82 (2.62, 3.01)	2.82 (2.68, 2.97)
	G/A (*n* = 39)	2.30 (1.67, 2.92)	2.88 (2.26, 3.50)	2.51 (2.06, 2.95)
Orientation	G/G (*n* = 387)	5.75 (5.59, 5.90) *	5.66 (5.50, 5.82)	5.70 (5.58, 5.82)
	G/A (*n* = 39)	5.15 (4.66, 5.63) *	5.73 (5.21, 6.25)	5.35 (4.98, 5.72)
MoCA score	G/G (*n* = 387)	24.80 (24.06, 25.54) *	24.61 (23.84, 25.38)	24.72 (24.17, 25.27)
	G/A (*n* = 39)	21.95 (19.63, 24.27) *	24.61 (22.14, 27.08)	22.92 (21.18, 24.67)

Participants were classified according to categories of MTHFR G1793A genotypes and serum vitamin B_12_ concentration. The data were presented as means (95% CI). General linear model was used for the analysis. Factors including gender, age, BMI, education, living condition, reading and smoking habit were adjusted during data analysis. MoCA, Montreal Cognitive Assessment; MTHFR, methylene tetrahydrofolate reductase. *p* value < 0.05 was considered statistically significant with asterisk superscript (*): *p* < 0.05.

**Table 7:** MoCA scores by serum Hcy level and MTHFR G1793A genotypes.

Cognition	Genotypes	Hcy (μmol/L)		All Subjects
		≤11.9	>11.9	
Visual-spatial and executive ability	G/G (*n* = 387)	3.64 (3.46, 3.82)	3.61 (3.44, 3.77)	3.62 (3.49, 3.75)
	G/A (*n* = 39)	3.56 (3.02, 4.11)	3.17 (2.59, 3.74)	3.36 (2.94, 3.77)
Name	G/G (*n* = 387)	2.83 (2.74, 2.93)	2.90 (2.84, 2.96)	2.87 (2.81, 2.93)
	G/A (*n* = 39)	2.90 (2.84, 2.96)	2.75 (2.54, 2.96)	2.75 (2.57, 2.93)
Attention	G/G (*n* = 387)	5.20 (4.99, 5.41)	5.07 (4.91, 5.23)	5.14 (5.01, 5.28)
	G/A (*n* = 39)	5.93 (4.30, 5.56)	5.03 (4.46, 5.60)	4.82 (4.40, 5.24)
Language	G/G (*n* = 387)	2.09 (1.97, 2.21)	2.08 (1.97, 2.19)	2.07 (1.99, 2.16)
	G/A (*n* = 39)	2.01 (1.66, 2.36)	1.91 (1.52, 2.30)	1.93 (1.66, 2.20)
Abstraction	G/G (*n* = 387)	1.58 (1.48, 1.68)	1.55 (1.45, 1.65)	1.57 (1.50, 1.64) *
	G/A (*n* = 39)	1.27 (0.96, 1.58)	1.34 (1.01, 1.68)	1.35 (1.09, 1.55) *
Delayed memory	G/G (*n* = 387)	2.89 (2.69, 3.09)	2.71 (2.52, 2.90)	2.82 (2.68, 2.97)
	G/A (*n* = 39)	2.68 (2.08, 3.29)	2.55 (1.89, 3.22)	2.51 (2.06, 2.95)
Orientation	G/G (*n* = 387)	5.61 (5.43, 5.80)	5.78 (5.66, 5.91)	5.70 (5.58, 5.82)
	G/A (*n* = 39)	5.42 (4.86, 5.99)	5.52 (5.10, 5.95)	5.35 (4.98, 5.72)
MoCA score	G/G (*n* = 387)	24.74 (23.86, 25.61)	24.64 (24.03, 25.26)	24.72 (24.17, 25.28)
	G/A (*n* = 39)	23.60 (20.97, 26.22)	23.18 (21.05, 25.31)	22.92 (21.18, 24.67)

Participants were classified according to MTHFR G1793A genotypes and serum Hcy concentration. The Data were presented as mean (95% CI). General linear model was applied for the analysis. Factors including gender, age, BMI, education, living condition, reading and smoking habit were adjusted during data analysis. Hcy, homocysteine; MoCA, Montreal Cognitive Assessment; MTHFR, methylene tetrahydrofolate reductase. *p* value < 0.05 was considered statistically significant with asterisk superscript (*): *p* < 0.05.

**Table 8:** MoCA scores by SLC19A1 G80A genotypes.

Cognition	Genotypes	Scores
Visual-spatial and executive ability	G/G (*n* = 145)	3.48 (3.27, 3.70)
	G/A (*n* = 214)	3.68 (3.51, 3.86)
	A/A (*n* = 67)	3.55 (3.23, 3.87)
Name	G/G (*n* = 105)	2.84 (2.74, 2.93)
	G/A (*n* = 214)	2.87 (2.79, 2.95)
	A/A (*n* = 67)	2.87 (2.73, 3.02)
Attention	G/G (*n* = 105)	5.01 (4.79, 5.23)
	G/A (*n* = 214)	5.19 (5.00, 5.37)
	A/A (*n* = 67)	5.10 (4.77, 5.43)
Language	G/G (*n* = 105)	2.00 (1.86, 2.14)
	G/A (*n* = 214)	2.06 (1.94, 2.18)
	A/A (*n* = 67)	2.19 (1.98, 2.40)
Abstraction	G/G (*n* = 105)	1.52 (1.40, 1.64)
	G/A (*n* = 214)	1.54 (1.44, 1.64)
	A/A (*n* = 67)	1.65 (1.47, 1.83)
Delayed memory	G/G (*n* = 105)	2.55 (2.23, 2.77) ^a^
	G/A (*n* = 214)	2.88 (2.69, 3.07) ^b^
	A/A (*n* = 67)	3.04 (2.70, 3.38) ^b^
Orientation	G/G (*n* = 105)	5.66 (5.46, 5.85)
	G/A (*n* = 214)	5.65 (5.49, 5.81)
	A/A (*n* = 67)	5.76 (5.47, 6.05)
MoCA score	G/G (*n* = 105)	23.98 (23.06, 24.90)
	G/A (*n* = 214)	24.77 (24.02, 25.53)
	A/A (*n* = 67)	25.09 (23.74, 26.44)

Participants were classified according to SLC19A1 G80A genotypes. The data were reported as means (95% CI). General linear model was utilized for the analyses of the differences in groups and Bonferroni correction for multiple comparisons. Factors including gender, age, BMI, education, living condition, reading and smoking habit were adjusted during data analysis. MoCA, Montreal Cognitive Assessment; SLC19A1, RFC (reduced folate carrier). *p* value < 0.05 was considered as significance. ^a,b^: significantly different for multiple comparisons among different SLC19A1 G80A genotypes, *p* < 0.05.

## Supplementary Materials

The following are available online at http://www.mdpi.com/2072-6643/8/10/665/s1, Table S1: MoCA scores by serum folate level and MTHFR C677T genotypes, Table S2: MoCA scores by serum vitamin B_12_ level and MTHFR C677T genotypes, Table S3: MoCA scores by serum Hcy level and MTHFR C677T genotypes, Table S4: MoCA scores by serum folate level and MTHFR A1298C genotypes, Table S5: MoCA scores by serum vitamin B_12_ level and MTHFR A1298C genotypes, Table S6: MoCA scores by serum Hcy level and MTHFR A1298C genotypes, Table S7: MoCA scores by serum folate level and SLC19A1 G80A genotypes, Table S8: MoCA scores by serum vitamin B_12_ level and SLC19A1 G80A genotypes, Table S9: MoCA scores by serum Hcy level and SLC19A1 G80A genotypes.
