# Supplementary material for: Association of MTHFR, SLC19A1 Genetic Polymorphism, Serum Folate, Vitamin B12 and Hcy Status with Cognitive Functions in Chinese Adults
Source: Nutrients. 2016 Oct 24;8(10):665. doi: 10.3390/nu8100665 (PMC5084051; doi:10.3390/nu8100665)
Supplement: Supplementary file 1 [file nutrients-08-00665-s001.docx]

Supplementary Materials: Association of MTHFR, SLC19A1 Genetic Polymorphism, Serum Folate, Vitamin B_12_ and Hcy Status with Cognitive Functions in Chinese Adults

Can Cai, Rong Xiao, Van Halm-Lutterodt Nicholas, Jie Zhen, Xiaochen Huang, Yao Xu, Shuying Chen and Linhong Yuan

Supporting information

**Table S1.** MoCA scores by serum folate level and MTHFR C677T genotypes.

| **Cognition** | **Genotypes** | **Folate, μg/mL** | | **All Subjects** |
| --- | --- | --- | --- | --- |
|  |  | **≤7.49** | **>7.49** |  |
| Visual-spatial and  executive ability | C/C (*n* = 67) | 3.56 (3.16, 3.95) | 3.43 (2.97, 3.88) | 3.55 (3.23, 3.86) |
|  | C/T (*n* = 199) | 3.62 (3.40, 3.84) | 3.63 (3.36, 3.90) | 3.58 (3.39, 3.77) |
|  | T/T (*n* = 160) | 3.67 (3.42, 3.92) | 3.57 (3.27, 3.87) | 3.63 (3.43, 3.84) |
| Name | C/C (*n* = 67) | 2.88 (2.76, 3.00) | 2.72 (2.48, 2.96) | 2.83 (2.79, 2.97) |
|  | C/T (*n* = 199) | 2.90 (2.83, 2.97) | 2.82 (2.68, 2.96) | 2.85 (2.77, 2.94) |
|  | T/T (*n* = 160) | 2.94 (2.86, 3.02) | 2.82 (2.66, 2.98) | 2.88 (2.79, 2.97) |
| Attention | C/C (*n* = 67) | 5.32 (4.94, 5.69) | 4.97 (4.45, 5.50) | 5.19 (4.87, 5.52) |
|  | C/T (*n* = 199) | 5.13 (4.92, 5.34) | 5.02 (4.71, 5.33) | 5.06 (4.87, 5.25) |
|  | T/T (*n* = 160) | 5.16 (4.92, 5.40) | 5.17 (4.82, 5.52) | 5.15 (4.94, 5.36) |
| Language | C/C (*n* = 67) | 2.07 (1.82, 2.33) | 1.85 (1.56, 2.15) | 1.96 (1.76, 2.17) |
|  | C/T (*n* = 199) | 2.20 (2.05, 2.34) | 2.02 (1.84, 2.19) | 2.07 (1.95, 2.19) |
|  | T/T (*n* = 160) | 2.12 (1.96, 2.28) | 2.03 (1.84, 2.23) | 2.09 (1.96, 2.23) |
| Abstraction | C/C (*n* = 67) | 1.55 (1.33, 1.77) | 1.41 (1.15, 1.68) | 1.51 (1.33, 1.68) |
|  | C/T (*n* = 199) | 1.62 (1.50, 1.75) | 1.36 (1.20, 1.52) | 1.49 (1.39, 1.59) |
|  | T/T (*n* = 160) | 1.73 (1.59, 1.87) | 1.52 (1.34, 1.69) | 1.63 (1.52, 1.75) |
| Delayed memory | C/C (*n* = 67) | 3.10 (2.64, 3.55) | 2.69 (2.20, 3.18) | 2.89 (2.55, 3.23) |
|  | C/T (*n* = 199) | 2.91 (2.66, 3.17) | 2.62 (2.33, 2.91) | 2.75 (2.55, 2.95) |
|  | T/T (*n* = 160) | 2.93 (2.65, 3.22) | 2.57 (2.24, 2.89) | 2.81 (2.59, 3.03) |
| Orientation | C/C (*n* = 67) | 5.77 (5.53, 6.01) | 5.32 (4.84, 5.80) | 5.59 (5.31, 3.87) |
|  | C/T (*n* = 199) | 5.85 (5.72, 5.99) | 5.50 (5.22, 5.79) | 5.64 (5.48, 5.81) |
|  | T/T (*n* = 160) | 5.86 (5.70, 6.01) | 5.61 (5.29, 5.93) | 5.73 (5.55, 5.92) |
| MoCA score | C/C (*n* = 67) | 25.11 (23.78, 26.44) | 23.27 (21.07, 25.48) | 24.39 (23.06, 25.72) |
|  | C/T (*n* = 199) | 25.16 (24.41, 25.91) | 23.88 (22.58, 25.18) | 24.36 (23.58, 25.15) |
|  | T/T (*n* = 160) | 25.36 (24.51, 26.21) | 24.21 (22.75, 25.66) | 24.86 (23.99, 25.72) |

Participants were classified according to categories of MTHFR C677T genotypes and median folate concentration. The Data are presented as the mean (95% CI). General linear model was applied for the analysis, adjusted for gender, age, BMI, education, living condition, reading and smoking habit. MoCA, Montreal Cognitive Assessment; MTHFR, methylene tetrahydrofolate reductase. *p* value < 0.05 was considered as significance.

**Table S2.** MoCA scores by serum vitamin B_12_ level and MTHFR C677T genotypes.

| **Cognition** | **Genotypes** | **Vitamin B_12_, pg/mL** | | **All Subjects** |
| --- | --- | --- | --- | --- |
|  |  | **≤412.5** | **>412.5** |  |
| Visual-spatial and  executive ability | C/C (*n* = 67) | 3.61 (3.19, 4.03) | 3.28 (2.83, 3.73) | 3.55 (3.23, 3.86) |
|  | C/T (*n* = 199) | 3.66 (3.39, 3.92) | 3.58 (3.35, 3.82) | 3.58 (3.39, 3.77) |
|  | T/T (*n* = 160) | 3.56 (3.29, 3.83) | 3.74 (3.45, 4.02) | 3.63 (3.43, 3.84) |
| Name | C/C (*n* = 67) | 2.94 (2.76, 3.12) | 2.64 (2.44, 2.84) | 2.83 (2.79, 2.97) |
|  | C/T (*n* = 199) | 2.81 (2.70, 2.93) | 2.91 (2.80, 3.01) | 2.85 (2.77, 2.94) |
|  | T/T (*n* = 160) | 2.87 (2.75, 2.99) | 2.88 (2.75, 3.01) | 2.88 (2.79, 2.97) |
| Attention | C/C (*n* = 67) | 5.35 (4.91, 5.79) | 4.87 (4.40, 5.35) | 5.19 (4.87, 5.52) |
|  | C/T (*n* = 199) | 5.00 (4.72, 5.28) | 5.15 (4.90, 5.40) | 5.06 (4.87, 5.25) |
|  | T/T (*n* = 160) | 5.01 (4.72, 5.30) | 5.31 (5.01, 5.61) | 5.15 (4.94, 5.36) |
| Language | C/C (*n* = 67) | 1.98 (1.71, 2.25) | 1.90 (1.61, 2.19) | 1.96 (1.76, 2.17) |
|  | C/T (*n* = 199) | 2.15 (1.98, 2.32) | 2.05 (1.90, 2.20) | 2.07 (1.95, 2.19) |
|  | T/T (*n* = 160) | 2.13 (1.95, 2.31) | 2.07 (1.88, 2.25) | 2.09 (1.96, 2.23) |
| Abstraction | C/C (*n* = 67) | 1.55 (1.31, 1.79) | 1.44 (1.18, 1.69) | 1.51 (1.33, 1.68) |
|  | C/T (*n* = 199) | 1.49 (1.34, 1.64) | 1.48 (1.35, 1.62) | 1.49 (1.39, 1.59) |
|  | T/T (*n* = 160) | 1.62 (1.47, 1.78) | 1.63 (1.46, 1.79) | 1.63 (1.52, 1.75) |
| Delayed memory | C/C (*n* = 67) | 2.82 (2.35, 3.28) | 3.01 (2.51, 3.51) | 2.89 (2.55, 3.23) |
|  | C/T (*n* = 199) | 2.73 (2.44, 3.02) | 2.74 (2.47, 3.00) | 2.75 (2.54, 2.95) |
|  | T/T (*n* = 160) | 2.71 (2.41, 3.01) | 2.88 (2.56, 3.19) | 2.81 (2.59, 3.03) |
| Orientation | C/C (*n* = 67) | 5.78 (5.41, 6.14) | 5.25 (4.83, .5,66) | 5.59 (5.31, 3.87) |
|  | C/T (*n* = 199) | 5.62 (5.39, 5.85) | 5.73 (5.51, 5.95) | 5.64 (5.48, 5.81) |
|  | T/T (*n* = 160) | 5.73 (5.49, 5.97) | 5.75 (5.48, 6.01) | 5.73 (5.55, 5.92) |
| MoCA score | C/C (*n* = 67) | 24.88 (23.15, 26.61) | 23.29 (21.31, 25.27) | 24.39 (23.06, 25.72) |
|  | C/T (*n* = 199) | 24.36 (23.26, 25.46) | 24.57 (23.53, 25.60) | 24.36 (23.58, 25.15) |
|  | T/T (*n* = 160) | 24.56 (23.26, 25.69) | 25.19 (23.94, 26.45) | 24.86 (23.99, 25.72) |

Participants were classified according to categories of MTHFR C677T genotypes and median vitamin B_12_ concentration. The Data are presented as the mean (95% CI). General linear model was applied for the analysis, adjusted for gender, age, BMI, education, living condition, reading and smoking habit. MoCA, Montreal Cognitive Assessment; MTHFR, methylene tetrahydrofolate reductase. *p* value < 0.05 was considered as significance.

**Table S3.** MoCA scores by serum Hcy level and MTHFR C677T genotypes.

| **Cognition** | **Genotypes** | **Hcy, μmol/L** | | **All Subjects** |
| --- | --- | --- | --- | --- |
|  |  | **≤11.9** | **>11.9** |  |
| Visual-spatial and  executive ability | C/C (*n* = 67) | 3.47 (3.04, 3.90) | 3.53 (3.10, 3.95) | 3.55 (3.23, 3.86) |
|  | C/T (*n* = 199) | 3.67 (3.43, 3.91) | 3.53 (3.28, 3.78) | 3.58 (3.39, 3.77) |
|  | T/T (*n* = 160) | 3.66 (3.35, 4.00) | 3.62 (3.38, 3.86) | 3.63 (3.43, 3.84) |
| Name | C/C (*n* = 67) | 2.77 (2.55, 2.99) | 2.86 (2.71, 3.02) | 2.83 (2.79, 2.97) |
|  | C/T (*n* = 199) | 2.83 (2.71. 2.96) | 2.90 (2.81, 2.99) | 2.85 (2.77, 2.94) |
|  | T/T (*n* = 160) | 2.86 (2.70, 3.02) | 2.89 (2.80, 2.98) | 2.88 (2.79, 2.97) |
| Attention | C/C (*n* = 67) | 5.25 (4.76, 5.75) | 5.12 (4.70, 5.53) | 5.19 (4.87, 5.52) |
|  | C/T (*n* = 199) | 5.12 (4.84, 5.40) | 5.01 (4.76, 5.25) | 5.06 (4.87, 5.25) |
|  | T/T (*n* = 160) | 5.21 (4.85, 5.57) | 5.10 (4.86, 5.34) | 5.15 (4.94, 5.36) |
| Language | C/C (*n* = 67) | 2.11 (1.83, 2.38) | 1.80 (1.51, 2.08) | 1.96 (1.76, 2.17) |
|  | C/T (*n* = 199) | 2.09 (1.93, 2.24) | 2.12 (1.96, 2.29) | 2.07 (1.95, 2.19) |
|  | T/T (*n* = 160) | 2.06 (1.86, 2.26) | 2.10 (1.94, 2.26) | 2.09 (1.96, 2.23) |
| Abstraction | C/C (*n* = 67) | 1.66 (1.42, 1.91) | 1.29 (1.04, 1.53) | 1.51 (1.33, 1.68) |
|  | C/T (*n* = 199) | 1.45 (1.31, 1.59) | 1.54 (1.40, 1.58) | 1.49 (1.39, 1.59) |
|  | T/T (*n* = 160) | 1.65 (1.47,1.83) | 1.61 (1.47, 1.75) | 1.63 (1.52, 1.75) |
| Delayed memory | C/C (*n* = 67) | 3.17 (2.70, 3.65) | 2.54 (2.05, 3.03) | 2.89 (2.55, 3.23) |
|  | C/T (*n* = 199) | 2.75 (2.48, 3.02) | 2.77 (2.48, 3.05) | 2.75 (2.55, 2.95) |
|  | T/T (*n* = 160) | 2.90 (2.55, 3.25) | 2.68 (2.40, 2.98) | 2.81 (2.59, 3.03) |
| Orientation | C/C (*n* = 67) | 5.47 (5.02, 5.92) | 5.68 (5.36, 5.99) | 5.59 (5.31, 3.87) |
|  | C/T (*n* = 199) | 5.63 (5.38, 5.88) | 5.74 (5.56, 5.92) | 5.64 (5.48, 5.81) |
|  | T/T (*n* = 160) | 5.61 (5.28, 5.93) | 5.81 (5.63, 5.99) | 5.73 (5.55, 5.92) |
| MoCA score | C/C (*n* = 67) | 24.79 (22.72, 26.87) | 23.69 (22.11, 25.27) | 24.39 (23.06, 25.72) |
|  | C/T (*n* = 199) | 24.44 (23.27, 25.60) | 24.55 (23.62, 25.47) | 24.36 (23.58, 25.15) |
|  | T/T (*n* = 160) | 24.83 (23.31, 26.34) | 24.79 (23.89, 25.69) | 24.86 (23.99, 25.72) |

Participants were classified according to categories of MTHFR C677T genotypes and median Hcy concentration. The Data are presented as the mean (95% CI). General linear model was applied for the analysis, adjusted for gender, age, BMI, education, living condition, reading and smoking habit. MoCA, Montreal Cognitive Assessment; MTHFR, methylene tetrahydrofolate reductase. *p* value < 0.05 was considered as significance.

**Table S4.** MoCA scores by serum folate level and MTHFR A1298C genotypes.

| **Cognition** | **Genotypes** | **Folate, μg/mL** | |
| --- | --- | --- | --- |
|  |  | **≤7.49** | **>7.49** |
| Visual-spatial and executive ability | A/A (*n* = 326) | 3.67 (3.30, 3.84) | 3.61 (3.40, 3.81) |
|  | A/C + C/C (*n* = 100) | 3.49 (3.17, 3.81) | 3.48 (3.10, 3.85) |
| Name | A/A (*n* = 326) | 2.93 (2.87, 2.98) | 2.78 (2.67, 2.89) |
|  | A/C + C/C (*n* = 100) | 2.86 (2.76, 2.96) | 2.88 (2.68, 3.08) |
| Attention | A/A (*n* = 326) | 5.15 (4.99, 5.32) | 5.04 (4.80, 5.28) |
|  | A/C + C/C (*n* = 100) | 5.22 (4.91, 5.52) | 5.16 (5.72, 5.59) |
| Language | A/A (*n* = 326) | 2.15 (2.04, 2.26) | 2.02 (1.88, 2.15) |
|  | A/C + C/C (*n* = 100) | 2.14 (1.94, 2.35) | 1.92 (1.68, 2.17) |
| Abstraction | A/A (*n* = 326) | 1.67 (1.58, 1.77) | 1.46 (1.34, 1.59) |
|  | A/C + C/C (*n* = 100) | 1.57 (1.40, 1.75) | 1.31 (1.09, 1.53) |
| Delayed memory | A/A (*n* = 326) | 2.91 (2.72, 3.11) | 2.64 (2.41, 2.86) |
|  | A/C + C/C (*n* = 100) | 3.07 (2.70, 3.44) | 2.53 (2.13, 2.94) |
| Orientation | A/A (*n* = 326) | 5.85 (5.75, 5.96) | 5.51 (5.28, 5.73) |
|  | A/C + C/C (*n* = 100) | 5.81 (5.61, 6.00) | 5.53 (5.13, 5.93) |
| MoCA score | A/A (*n* = 326) | 25.28 (24.70, 25.87) | 23.96 (22.95, 24.97) |
|  | A/C + C/C (*n* = 100) | 25.04 (23.96, 26.12) | 23.74 (21.93, 25.55) |

Participants were classified according to categories of MTHFR A1298C genotypes and median folate concentration. The Data are presented as the mean (95% CI). General linear model was applied for the analysis, adjusted for gender, age, BMI, education, living condition, reading and smoking habit. MoCA, Montreal Cognitive Assessment; MTHFR, methylene tetrahydrofolate reductase. *p* value < 0.05 was considered as significance.

**Table S5.** MoCA scores by serum vitamin B_12_ level and MTHFR A1298C genotypes.

| **Cognition** | **Genotypes** | **Vitamin B_12_, pg/mL** | |
| --- | --- | --- | --- |
|  |  | **≤412.5** | **>412.5** |
| Visual-spatial and executive ability | A/A (*n* = 326) | 3.63 (3.44, 3.82) | 3.65 (3.46, 3.84) |
|  | A/C + C/C (*n* = 100) | 3.53 (3.17, 3.90) | 2.42 (3.08, 3.75) |
| Name | A/A (*n* = 326) | 2.84 (2.75, 2.92) | 2.88 (2.79, 2.96) |
|  | A/C + C/C (*n* = 100) | 2.93 (2.77, 2.96) | 2.82 (2.66, 2.97) |
| Attention | A/A (*n* = 326) | 5.05 (4.84, 5.25) | 5.16 (4.96, 5.36) |
|  | A/C + C/C (*n* = 100) | 5.13 (4.74, 5.52) | 5.19 (4.83, 5.55) |
| Language | A/A (*n* = 326) | 2.13 (2.00, 2.26) | 2.05 (1.93, 2.17) |
|  | A/C + C/C (*n* = 100) | 2.05 (1.81, 2.30) | 2.00 (1.78, 2.22) |
| Abstraction | A/A (*n* = 326) | 1.57 (1.46, 1.69) | 1.57 (1.46, 1.68) |
|  | A/C + C/C (*n* = 100) | 1.49 (1.28, 1.70) | 1.39 (1.20, 1.58) |
| Delayed memory | A/A (*n* = 326) | 2.70 (2.48, 2.91) | 2.86 (2.65, 3.08) |
|  | A/C + C/C (*n* = 100) | 2.87 (2.46, 3.28) | 2.71 (2.33, 3.08) |
| Orientation | A/A (*n* = 326) | 5.67 (5.50, 5.84) | 5.71 (5.53, 5.88) |
|  | A/C + C/C (*n* = 100) | 5.78 (5.46, 6.10) | 5.55 (5.23, 5.86) |
| MoCA score | A/A (*n* = 326) | 24.51 (23.70, 25.32) | 24.80 (23.96, 25.65) |
|  | A/C + C/C (*n* = 100) | 24.62 (23.10, 26.15) | 24.01 (22.51, 25.51) |

Participants were classified according to categories of MTHFR A1298C genotypes and median vitamin B_12_ concentration. The Data are presented as the mean (95% CI). General linear model was applied for the analysis, adjusted for gender, age, BMI, education, living condition, reading and smoking habit. MoCA, Montreal Cognitive Assessment; MTHFR, methylene tetrahydrofolate reductase. *p* value < 0.05 was considered as significance.

**Table S6.** MoCA scores by serum Hcy level and MTHFR A1298C genotypes.

| **Cognition** | **Genotypes** | **Hcy, μmol/L** | |
| --- | --- | --- | --- |
|  |  | **≤11.9** | **>11.9** |
| Visual-spatial and executive ability | A/A (*n* = 326) | 3.67 (3.47, 3.88) | 3.62 (3.44, 3.79) |
|  | A/C + C/C (*n* = 100) | 3.53 (3.20, 3.85) | 3.37 (3.00, 3.74) |
| Name | A/A (*n* = 326) | 2.81 (2.70, 2.91) | 2.90 (2.83, 2.96) |
|  | A/C + C/C (*n* = 100) | 2.89 (2.72, 3.06) | 2.84 (2.71, 2.98) |
| Attention | A/A (*n* = 326) | 5.12 (4.88, 5.35) | 5.08 (4.91, 5.26) |
|  | A/C + C/C (*n* = 100) | 5.31 (4.93, 5.68) | 4.99 (4.63, 5.36) |
| Language | A/A (*n* = 326) | 2.06 (1.93, 2.19) | 2.11 (1.99, 2.23) |
|  | A/C + C/C (*n* = 100) | 2.14 (1.93, 2.35) | 1.89 (1.64, 2.14) |
| Abstraction | A/A (*n* = 326) | 1.59 (1.47, 1.70) | 1.56 (1.46, 1.67) |
|  | A/C + C/C (*n* = 100) | 1.45 (1.26, 1.64) | 1.40 (1.19, 1.62) |
| Delayed memory | A/A (*n* = 326) | 2.89 (2.66, 3.12) | 2.67 (2.47, 2.88) |
|  | A/C + C/C (*n* = 100) | 2.81 (2.45, 3.18) | 2.80 (2.37, 3.23) |
| Orientation | A/A (*n* = 326) | 5.58 (5.37, 5.80) | 5.77 (5.64, 5.90) |
|  | A/C + C/C (*n* = 100) | 5.62 (5.28, 5.97) | 5.72 (5.45, 6.00) |
| MoCA score | A/A (*n* = 326) | 24.60 (23.62, 25.59) | 24.67 (24.01, 25.33) |
|  | A/C + C/C (*n* = 100) | 24.66 (23.08, 26.24) | 23.92 (22.54, 25.30) |

Participants were classified according to categories of MTHFR A1298C genotypes and median Hcy concentration. The Data are presented as the mean (95% CI). General linear model was applied for the analysis, adjusted for gender, age, BMI, education, living condition, reading and smoking habit. MoCA, Montreal Cognitive Assessment; MTHFR, methylene tetrahydrofolate reductase. *p* value < 0.05 was considered as significance.

**Table S7.** MoCA scores by serum folate level and SLC19A1 G80A genotypes.

| **Cognition** | **Genotypes** | **Folate, μg/mL** | |
| --- | --- | --- | --- |
|  |  | **≤7.49** | **>7.49** |
| Visual-spatial and executive ability | G/G (*n* = 145) | 3.58 (3.31, 3.86) | 3.39 (3.09, 3.69) |
|  | G/A (*n* = 214) | 3.72 (3.50, 3.94) | 3.68 (3.42, 3.94) |
|  | A/A (*n* = 67) | 3.45 (3.09, 3.81) | 3.68 (3.18, 4.18) |
| Name | G/G (*n* = 105) | 2..89 (2.80, 2.97) | 2.75 (2.60, 2.91) |
|  | G/A (*n* = 214) | 2.95 (2.88, 3.02) | 2.82 (2.69, 2.96) |
|  | A/A (*n* = 67) | 2.85 (2.74, 2.96) | 2.88 (2.62, 3.15) |
| Attention | G/G (*n* = 105) | 5.07 (4.81, 5.34) | 4.95 (4.60, 5.29) |
|  | G/A (*n* = 214) | 5.24 (5.03, 5.45) | 5.23 (4.93, 5.52) |
|  | A/A (*n* = 67) | 5.13 (4.78, 5.47) | 4.80 (4.22, 5.38) |
| Language | G/G (*n* = 105) | 2.07 (1.89, 2.25) | 1.94 (1.74, 2.13) |
|  | G/A (*n* = 214) | 2.18 (2.04, 2.32) | 2.00 (1.84, 2.17) |
|  | A/A (*n* = 67) | 2.20 (1.97, 2.44) | 2.14 (1.81, 2.46) |
| Abstraction | G/G (*n* = 105) | 1.65 (1.49, 1.80) | 1.38 (1.21, 1.56) |
|  | G/A (*n* = 214) | 1.62 (1.50, 1.75) | 1.45 (1.30, 1.60) |
|  | A/A (*n* = 67) | 1.74 (1.53, 1.94) | 1.45 (1.16, 1.75) |
| Delayed memory | G/G (*n* = 105) | 2.65 (2.34, 2.96) | 2.40 (2.08, 2.73) |
|  | G/A (*n* = 214) | 3.09 (2.84, 3.34) | 2.71 (2.43, 2.99) |
|  | A/A (*n* = 67) | 3.08 (2.67, 3.49) | 2.83 (2.29, 3.38) |
| Orientation | G/G (*n* = 105) | 5.80 (5.63, 5.97) | 5.47 (5.15, 5.79) |
|  | G/A (*n* = 214) | 5.84 (5.71, 5.97) | 5.53 (5.26, 5.81) |
|  | A/A (*n* = 67) | 5.92 (5.70, 6.14) | 5.56 (5.02, 6.10) |
| MoCA score | G/G (*n* = 105) | 24.64 (23.71, 25.56) | 23.20 (21.75, 24.66) |
|  | G/A (*n* = 214) | 25.56 (24.83, 26.29) | 24.34 (23.09, 25.59) |
|  | A/A (*n* = 67) | 25.33 (24.11, 26.54) | 24.22 (21.78, 26.66) |

Participants were classified according to categories of SLC19A1 G80A genotypes and median folate concentration. The Data are presented as the mean (95% CI). General linear model was applied for the analysis, adjusted for gender, age, BMI, education, living condition, reading and smoking habit. MoCA, Montreal Cognitive Assessment; MTHFR, methylene tetrahydrofolate reductase. *p* value < 0.05 was considered as significance.

**Table S8.** MoCA scores by serum vitamin B_12_ level and SLC19A1 G80A genotypes.

| **Cognition** | **Genotypes** | **Vitamin B_12_, pg/mL** | |
| --- | --- | --- | --- |
|  |  | **≤412.5** | **>412.5** |
| Visual-spatial and executive ability | G/G (*n* = 145) | 3.43 (3.16, 3.70) | 3.53 (3.21, 3.84) |
|  | G/A (*n* = 214) | 3.81 (3.55, 4.06) | 3.61 (3.38, 3.83) |
|  | A/A (*n* = 67) | 3.52 (3.10, 3.94) | 3.69 (3.25, 4.13) |
| Name | G/G (*n* = 105) | 2.82 (2.70, 2.94) | 2.83 (2.68, 2.97) |
|  | G/A (*n* = 214) | 2.87 (2.76, 2.99) | 2.89 (2.78, 2.99) |
|  | A/A (*n* = 67) | 2.91 (2.73, 3.10) | 2.83 (2.63, 3.03) |
| Attention | G/G (*n* = 105) | 4.99 (4.70, 5.28) | 5.04 (4.71, 5.37) |
|  | G/A (*n* = 214) | 5.13 (4.85, 5.41) | 5.28 (5.04, 5.51) |
|  | A/A (*n* = 67) | 5.08 (4.63, 5.52) | 4.99 (4.53, 5.46) |
| Language | G/G (*n* = 105) | 2.05 (1.87, 2.23) | 1.92 (1.72, 2.12) |
|  | G/A (*n* = 214) | 2.06 (1.89, 2.23) | 2.11 (1.97, 2.25) |
|  | A/A (*n* = 67) | 2.40 (2.13, 2.68) | 1.97 (1.69, 2.25) |
| Abstraction | G/G (*n* = 105) | 1.51 (1.35, 1.67) | 1.49 (1.32, 1.67) |
|  | G/A (*n* = 214) | 1.56 (1.41, 1.71) | 1.53 (1.40, 1.65) |
|  | A/A (*n* = 67) | 1.64 (1.39, 1.71) | 1.60 (1.35, 1.85) |
| Delayed memory | G/G (*n* = 105) | 2.47 (2.17, 2.77) | 2.59 (2.24, 2.94) |
|  | G/A (*n* = 214) | 2.85 (2.56, 3.13) | 2.92 (2.67, 3.17) |
|  | A/A (*n* = 67) | 3.07 (2.61, 3.53) | 2.93 (2.44, 3.41) |
| Orientation | G/G (*n* = 105) | 5.63 (5.39, 5.87) | 5.62 (5.33, 5.92) |
|  | G/A (*n* = 214) | 5.65 (5.43, 5.88) | 5.69 (5.48, 5.90) |
|  | A/A (*n* = 67) | 5.94 (5.57, 6.30) | 5.66 (5.25, 6.07) |
| MoCA score | G/G (*n* = 105) | 23.84 (22.71, 24.98) | 23.93 (22.56, 25.31) |
|  | G/A (*n* = 214) | 24.80 (23.72, 25.88) | 24.96 (23.97, 25.95) |
|  | A/A (*n* = 67) | 25.50 (23.75, 27.24) | 24.58 (22.64, 26.52) |

Participants were classified according to categories of SLC19A1 G80A genotypes and median vitamin B_12_ concentration. The Data are presented as the mean (95% CI). General linear model was applied for the analysis, adjusted for gender, age, BMI, education, living condition, reading and smoking habit. MoCA, Montreal Cognitive Assessment; MTHFR, methylene tetrahydrofolate reductase. *p* value < 0.05 was considered as significance.

**Table S9.** MoCA scores by serum Hcy level and SLC19A1 G80A genotypes.

| **Cognition** | **Genotypes** | **Hcy, μmol/L** | |
| --- | --- | --- | --- |
|  |  | **≤11.9** | **>11.9** |
| Visual-spatial and executive ability | G/G (*n* = 145) | 3.47 (3.16, 3.78) | 3.47 (3.21, 3.74) |
|  | G/A (*n* = 214) | 3.67 (3.43, 3.90) | 3.72 (3.48, 3.96) |
|  | A/A (*n* = 67) | 3.86 (3.40, 4.32) | 3.38 (2.99, 3.77) |
| Name | G/G (*n* = 105) | 2.85 (2.69, 3.01) | 2.80 (2.71, 2.90) |
|  | G/A (*n* = 214) | 2.83 (2.71, 2.95) | 2.95 (2.86, 3.04) |
|  | A/A (*n* = 67) | 2.81 (2.57, 3.05) | 2.91 (2.77, 3.05) |
| Attention | G/G (*n* = 105) | 5.06 (4.70, 5.42) | 4.97 (4.71, 5.23) |
|  | G/A (*n* = 214) | 5.29 (5.02, 5.56) | 5.16 (4.92, 5.39) |
|  | A/A (*n* = 67) | 4.94 (4.41, 5.47) | 5.05 (4.66, 5.43) |
| Language | G/G (*n* = 105) | 2.02 (1.82, 2.22) | 1.97 (1.80, 2.15) |
|  | G/A (*n* = 214) | 2.12 (1.97, 2.27) | 2.06 (1.90, 2.22) |
|  | A/A (*n* = 67) | 2.05 (1.76, 2.35) | 2.29 (2.03, 2.55) |
| Abstraction | G/G (*n* = 105) | 1.48 (1.29, 1.66) | 1.52 (1.36, 1.67) |
|  | G/A (*n* = 214) | 1.58 (1.45, 1.71) | 1.50 (1.36, 1.64) |
|  | A/A (*n* = 67) | 1.58 (1.31, 1.85) | 1.66 (1.43, 1.88) |
| Delayed memory | G/G (*n* = 105) | 2.58 (2.23, 2.92) | 2.48 (2.18, 2.78) |
|  | G/A (*n* = 214) | 2.99 (2.74, 3.25) | 2.76 (2.48, 3.03) |
|  | A/A (*n* = 67) | 3.01 (2.49, 3.52) | 3.02 (2.57, 3.46) |
| Orientation | G/G (*n* = 105) | 5.59 (5.26, 5.91) | 5.66 (5.47, 5.86) |
|  | G/A (*n* = 214) | 5.58 (5.34, 5.82) | 5.81 (5.63, 5.99) |
|  | A/A (*n* = 67) | 5.68 (5.19, 6.16) | 5.84 (5.55, 6.13) |
| MoCA score | G/G (*n* = 105) | 23.91 (22.40, 25.42) | 23.84 (22.89, 24.81) |
|  | G/A (*n* = 214) | 24.96 (23.84, 26.08) | 24.87 (23.98, 25.76) |
|  | A/A (*n* = 67) | 24.81 (22.58, 27.04) | 25.11 (23.68, 26.55) |

Participants were classified according to categories of SLC19A1 G80A genotypes and median Hcy concentration. The Data are presented as the mean (95% CI). General linear model was applied for the analysis, adjusted for gender, age, BMI, education, living condition, reading and smoking habit. MoCA, Montreal Cognitive Assessment; MTHFR, methylene tetrahydrofolate reductase. *p* value < 0.05 was considered as significance.
